# Psychopathy Facets Differentially Predict Drug- and Alcohol-Related Problems Following Violent Injury

**DOI:** 10.3390/bs16091560

**Published:** 2026-09-03

**Authors:** Jessica J. James, Nicholas D. Thomson

**Affiliations:** Department of Social and Behavioral Sciences, Virginia Commonwealth University, Richmond, VA 23219, USA

**Keywords:** psychopathy, psychopathy facets, substance misuse, drug use, alcohol use, longitudinal, externalizing, violent injury

## Abstract

Psychopathy is a multidimensional personality construct associated with substantial personal, familial, and societal burden. Among the various forms of psychopathology that co-occur with psychopathy, substance misuse represents one of its most prominent comorbidities. Despite growing interest in the psychopathy–substance misuse association, most facet-level studies have been cross-sectional or have relied on lifetime indices of substance involvement, limiting understanding of whether distinct psychopathy facets uniquely predict subsequent substance-related problem severity. The present study prospectively examined whether the four psychopathy facets uniquely predicted six-month drug- and alcohol-related problem severity among adults recovering from a violent injury. Participants (N = 106) completed baseline assessments of the four psychopathy facets using the Self-Report Psychopathy Scale–Short Form (SRP-SF) and drug- and alcohol-related problem severity using the Drug Abuse Screening Test (DAST-10) and Alcohol Use Disorders Identification Test (AUDIT), with drug- and alcohol-related problem severity reassessed six months later. Separate multiple regression models examined whether the interpersonal, affective, lifestyle, and antisocial psychopathy facets uniquely predicted six-month drug- and alcohol-related problem severity after controlling for baseline drug and alcohol use severity, age, sex, race, posttraumatic stress disorder (PTSD), history of head injury, and the substantial covariance among psychopathy facets. Affective psychopathic traits uniquely predicted greater six-month drug-related problem severity, whereas antisocial psychopathic traits uniquely predicted greater six-month alcohol-related problem severity. The remaining psychopathy facets were not uniquely associated with either outcome. These findings suggest that the psychopathy facets most strongly associated with substance misuse in cross-sectional research may differ from those that uniquely predict the persistence of drug- and alcohol-related problems over time, underscoring the importance of prospective facet-level research to inform etiological models, risk assessment, and targeted prevention and intervention.

## 1. Introduction

Psychopathy is a multidimensional personality construct associated with substantial personal, familial, and societal burden, including criminal justice involvement, educational and occupational impairment, adverse physical and mental health outcomes, and substantial economic costs (De Brito et al., 2021). Psychopathy is characterized by a constellation of interpersonal, affective, lifestyle, and antisocial features including manipulativeness, deceitfulness, callousness and diminished remorse, impulsivity and irresponsibility, and persistent antisocial behavior (Hare, 2003; Hare & Neumann, 2008; Neumann et al., 2007). Importantly, the burden associated with psychopathic traits extends beyond its well-established association with antisocial and criminal behavior. Psychopathy frequently co-occurs with other forms of psychopathology, among which substance use disorders represent a particularly prominent comorbidity (Verona et al., 2018). Given the considerable harms associated with both psychopathy and substance misuse, identifying the psychopathic traits that confer vulnerability to persistent substance-related problems represents an important clinical and public-health priority.

The association between psychopathy and substance misuse has traditionally been conceptualized as reflecting a shared externalizing liability (Ellingson et al., 2018; Rutherford et al., 2016). Externalizing models conceptualize alcohol and drug dependence, antisocial behavior, impulsivity, and related forms of psychopathology as manifestations of a broader disposition toward behavioral disinhibition (Verona et al., 2018). Facet-level studies have generally reported the strongest associations between substance misuse and the lifestyle and antisocial dimensions of psychopathy. For example, these behavioral features have been associated with substance use disorder severity, reduced likelihood of abstinence, and the covariance between psychopathy and broader externalizing psychopathology across multiple indicators of substance-related pathology (Brieman et al., 2024; Vincent et al., 2024; Walsh et al., 2007). Consequently, research examining the psychopathy–substance misuse association has focused primarily on the behavioral dimensions of psychopathy, with comparatively less attention directed toward whether interpersonal and affective psychopathic traits make unique contributions to substance misuse.

Although behavioral dimensions have received the greatest attention in the psychopathy–substance misuse literature, the externalizing model does not necessarily account for all processes through which psychopathic traits may contribute to persistent substance-related problems. Contemporary neurobiological conceptualizations of psychopathy provide a complementary perspective, emphasizing the potential contribution of affective psychopathic traits through impairment in affective learning and sensitivity to adverse consequences. Within this framework, psychopathy is conceptualized, in part, as arising from dysfunction in neural systems that support the processing of affective information, resulting in impaired stimulus-reinforcement learning and a diminished capacity for distress and punishment cues to modify subsequent behavior (Blair, 2006, 2007, 2013, 2019). Rather than reflecting a generalized impairment in emotional experience, this account proposes that disturbance in affective learning limits the extent to which adverse consequences acquire sufficient emotional and motivational significance to modify future behavior. Consistent with this conceptualization, evidence from experimental studies and meta-analytic research indicates that individuals with psychopathic traits show impaired learning from painful aversive outcomes, alongside broader reductions in responsiveness to others’ distress and emotional cues (Atanassova et al., 2024; Burghart & Mier, 2022). More specifically, facet-level psychophysiological research indicates that, after accounting for covariance among the four psychopathy facets, higher affective psychopathic trait scores were uniquely associated with dampened sympathetic nervous system reactivity during explicit fear induction (Thomson, 2022). Collectively, this literature suggests that affective psychopathic traits may be particularly relevant to the persistence of substance-related problems by limiting the extent to which adverse consequences shape future behavior, especially in contexts where continued misuse occurs despite repeated and highly salient adverse consequences. However, whether affective psychopathic traits uniquely predict the prospective course of substance-related problems remains underexplored.

Despite growing evidence implicating affective processes in psychopathy, relatively little prospective research has examined whether distinct psychopathy facets differentially predict the subsequent course of drug- and alcohol-related problems. Consistent with broader reviews of the psychopathy–substance misuse literature (Ellingson et al., 2018), most facet-level studies have been cross-sectional or have relied on lifetime indices of substance involvement (Brieman et al., 2024; Vincent et al., 2024; Walsh et al., 2007), thereby limiting conclusions regarding whether different psychopathy facets uniquely predict subsequent substance-related problem severity beyond their association with existing substance misuse. Although psychopathy is associated with both drug and alcohol misuse, these forms of substance involvement differ in important social, legal, and environmental contexts that may shape the mechanisms through which psychopathic traits contribute to their persistence. Whereas illicit drug use is more likely to occur in environments characterized by acute physical risk, social marginalization, and criminal-legal consequences, problematic alcohol use often occurs within socially normative contexts (Lansford et al., 2023; Park et al., 2020; Scher et al., 2023; Volkow, 2021). These contextual differences may be particularly relevant in populations experiencing violent injury, given established associations between psychopathic traits and substance misuse with engagement in risky, impulsive, and criminal behaviors that may increase exposure to situations involving perpetrating and experiencing violence (Daigle et al., 2020; Lau et al., 2024; Vincent et al., 2024). Moreover, much of the existing literature on psychopathy and substance misuse has examined incarcerated populations, making violently injured populations a relatively understudied context in which to examine these associations. Examining psychopathic traits and substance-related severity prospectively in adults recovering from violent injuries may therefore provide important insight into whether facet-level associations observed in predominantly cross-sectional research with correctional samples extend to another high-risk population and predict the subsequent course of substance-related problems.

The present study prospectively examined whether the four psychopathy facets differentially predicted six-month drug and alcohol use severity, with drug- and alcohol-related outcomes examined separately, among adults recovering from a violent injury. This population provides a theoretically relevant context for examining these associations because psychopathic traits and substance misuse are both associated with violence-related risk, while adults recovering from a violent injury represent a distinct high-risk population that has received comparatively little attention in psychopathy research. Examining substance-related outcomes following violent injury also provides an opportunity to investigate whether psychopathic traits are prospectively associated with the subsequent course of substance-related problems in the context of significant adverse experiences. By simultaneously modeling all four psychopathy facets, the present study evaluated the unique associations of each dimension with six-month drug and alcohol use severity after accounting for baseline drug and alcohol misuse, demographic covariates, and the substantial covariance among the psychopathy facets. Consistent with externalizing accounts of the psychopathy–substance misuse association, lifestyle and antisocial facets were expected to demonstrate prospective associations with substance-related severity. However, emerging evidence implicating affective processes suggests that interpersonal and affective psychopathic traits may also contribute to substance-related problems through processes not fully captured by a broader externalizing liability. Findings regarding these dimensions have been inconsistent, with studies reporting positive, null, or negative associations, while prospective research remains limited, leaving an important gap in the literature. Thus, given inconsistent findings regarding the specificity and direction of these associations, no hypotheses were made regarding the prospective associations of interpersonal and affective psychopathic traits with drug- and alcohol-related severity.

## 2. Methods

### 2.1. Participants

The broader study enrolled 488 adults who received hospital treatment for violent injury. Of these, 229 (46.9%) participated in the six-month follow-up. Participants who did not participate in the six-month follow-up had higher baseline psychopathy facet scores, and higher baseline drug and alcohol use severity. No significant differences were observed in other variables. The present study included 106 adults with complete-case data at the six-month follow-up assessment. Participants ranged in age from 18 to 75 years (*M_age_* = 34.01, *SD* = 13.06) and were predominantly male (65%). Most participants identified as Black (81%), while the remaining 19% identified as another race. At baseline, 38% of participants met criteria for PTSD, and 37% reported a history of head injury. In terms of injury type, 39 participants (36.8%) sustained gunshot wounds, 28 (26.4%) sustained assault-related injuries without a weapon, 8 (7.5%) sustained injuries from blunt weapons (e.g., baseball bat), 4 (3.8%) sustained other types of violent injury, and 2 (1.9%) sustained stab wounds; injury type was unreported for 25 participants (23.6%).

### 2.2. Procedure

Participants were recruited from an urban Level 1 Trauma Center in Virginia through either the emergency department or inpatient services as part of a broader study examining retaliatory gun violence (R01CE003296, PI: Thomson). Individuals were eligible if they were over the age of 18 years, had experienced a violent injury, and were not incarcerated. Prior to providing consent, participants were informed that the study was conducted solely for research purposes and that participation would not affect their medical treatment. Participants received $160 in compensation for their participation. All study procedures were approved by the Virginia Commonwealth University Institutional Review Board, and the project was covered by a Certificate of Confidentiality issued by the Centers for Disease Control and Prevention.

### 2.3. Measures

#### 2.3.1. Psychopathy

Psychopathy was assessed using the Self-Report Psychopathy Scale–Short Form (Paulhus et al., 2016), a 29-item measure assessing the four facets of psychopathy. These facets include interpersonal traits (7 items; for example, “I have pretended to be someone else in order to get something”), affective traits (7 items; for example, “I don’t feel bad about hurting people”), lifestyle traits (7 items; for example, “I’ve often done something dangerous just for the thrill of it”), and antisocial traits (7 items; for example, “I have tricked someone into giving me money”). Participants responded to items using a 5-point Likert scale ranging from 1 (disagree strongly) to 5 (agree strongly). Facet scores were calculated such that higher values reflected greater levels of psychopathic traits. In the current sample, the SRP-SF demonstrated strong internal consistency overall and across facets (total scale α = .92; interpersonal α = .82; affective α = .78; lifestyle α = .82; antisocial α = .79).

#### 2.3.2. Drug Use

Drug use was assessed at baseline and six-month follow-up using the Drug Abuse Screening Test (DAST-10; Skinner, 1982). The DAST-10 is a 10-item self-report measure that screens for problems associated with drug use. At baseline, the DAST-10 assessed problems associated with drug use during the past 12 months. At six-month follow-up, items were administered with a six-month reference period to assess drug-related problems occurring since baseline. Items are scored dichotomously (Yes/No) and summed to yield a total score ranging from 0 to 10. Higher scores indicate greater severity of drug-related problems. The DAST-10 demonstrated good internal consistency in the current sample (α = .83).

#### 2.3.3. Alcohol Use

Alcohol use was assessed at baseline and six-month follow-up using the Alcohol Use Disorders Identification Test (AUDIT; Saunders et al., 1993), a 10-item self-report measure that identifies hazardous and harmful patterns of alcohol use. The AUDIT assesses alcohol consumption, symptoms of dependence, and alcohol-related problems. At baseline, the AUDIT assessed alcohol-related problems during the past 12 months. At six-month follow-up, items were administered with a six-month reference period to assess alcohol-related problems occurring since baseline. Items are scored from 0 to 4 and summed to yield a total score ranging from 0 to 40. Higher scores indicate greater severity of problematic alcohol use. The AUDIT demonstrated good internal consistency in the current sample (α = .89).

#### 2.3.4. Posttraumatic Stress Disorder (PTSD)

PTSD symptoms were assessed at baseline using the Posttraumatic Stress Disorder Checklist DSM-5 (PCL-5; Blevins et al., 2015). The PCL-5 is a 20-item self-report measure assessing DSM-5 PTSD symptoms. Items are scored on a 5-point Likert scale ranging from 0 (not at all) to 4 (extremely). Consistent with prior research, PTSD was coded dichotomously using the recommended clinical cutoff score (≥31), with higher scores indicating clinically significant PTSD symptoms. The PCL-5 demonstrated excellent internal consistency in the current sample (α = .94).

#### 2.3.5. History of Head Injury

History of head injury was assessed at baseline using a single self-report item asking participants whether they had ever sustained an injury to the head. Responses were coded dichotomously (0 = no, 1 = yes).

### 2.4. Analytic Plan

Descriptive statistics and zero-order correlations were examined for all study variables. Multiple linear regression analyses were conducted to examine the unique prospective associations between the four psychopathy facets and drug and alcohol use severity at six-month follow-up. Separate models were estimated for drug and alcohol use severity. Missing data were handled using complete-case analysis, such that participants with missing data on any variable in a given regression model were excluded from that analysis. Each model included the interpersonal, affective, lifestyle, and antisocial psychopathy facets, controlling for age, sex, race, PTSD, history of head injury, and baseline drug and alcohol use severity. Given the demographic and contextual characteristics of the recruited population, race was included to account for potential differences in substance-use severity by race within this violent-injury sample. Simultaneously modeling all four psychopathy facets allowed the unique association of each facet with six-month substance use severity to be evaluated after accounting for the substantial covariance among the psychopathy dimensions. Baseline drug and alcohol use severity were included to determine whether psychopathy facets accounted for unique variance in six-month substance use severity beyond baseline substance-related problems. DAST-10 and AUDIT total scores were treated as continuous indices of drug- and alcohol-related problem severity, respectively. Distributional characteristics were examined using descriptive statistics, skewness and kurtosis estimates, and frequency distributions. The DAST-10 and AUDIT scores demonstrated positive skewness (skewness = 2.08 and 1.75, respectively). Although transformations were considered, both outcomes were retained as untransformed continuous severity scores because the measures were intended to capture variation in the severity of substance-related problems and demonstrated meaningful variability across their observed score ranges. Sensitivity analyses were conducted to characterize the minimum overall effect size detectable with 80% power given the sample size and number of predictors in each regression model, assuming α = .05. All analyses were performed in R (R Core Team, 2025). Multicollinearity was assessed using variance inflation factors (VIFs).

## 3. Results

### 3.1. Correlations Between Primary Study Variables

Descriptive Statistics and zero-order correlations are presented in Table 1. The four psychopathy facets were moderately to strongly intercorrelated, *r*s = .65 to .76, *p*s < .001. Drug use severity at six-month follow-up was positively associated with all psychopathy facets, *r*s .35 to .43, *p*s < .01. In contrast, six-month alcohol use severity was positively associated only with the lifestyle facet, *r* = .35, *p* = .001, and was not significantly correlated with the interpersonal, affective, or antisocial facets. VIFs ranged from 1.15 to 4.32 across the regression models, indicating acceptable levels of multicollinearity.

### 3.2. Drug Use Severity

Drug use severity at six-month follow-up was examined using multiple linear regression, accounting for demographic characteristics (e.g., age, sex, race), PTSD, history of head injury, and baseline drug and alcohol use (see Table 2). The overall model was statistically significant, *F*(11,94) = 5.81, *p* < .001, *R*^2^ = .41. Among the four psychopathy facets, only the affective facet was significantly associated with greater drug use severity, *b* = 0.12, SE = 0.05, *p* = .011. Baseline drug use also remained a significant predictor, *b* = 0.39, SE = 0.09, *p* < .001. The remaining psychopathy facets, demographic variables, PTSD, history of head injury, and baseline alcohol use were not significant predictors, *p*s > .05.

### 3.3. Alcohol Use Severity

Alcohol use severity at six-month follow-up was examined using multiple linear regression among 84 participants with complete data for the analysis, accounting for demographic characteristics (e.g., age, sex, race), PTSD, history of head injury, and baseline drug and alcohol use (see Table 3). Of the 106 participants with complete follow-up data, 22 were excluded from the alcohol model because of missing data on one or more model variables, representing approximately 21% of the available follow-up sample. This reduction in sample size may limit the representativeness of the alcohol analytic sample; however, comparisons between participants included in the model and those excluded revealed no evidence of systematic differences across available data. The overall model was statistically significant, *F*(11,72) = 14.69, *p* < .001, *R*^2^ = .69. Among the four psychopathy facets, only the antisocial facet was significantly associated with greater alcohol use severity, *b* = 0.43, SE = 0.14, *p* = .003. Baseline alcohol use, *b* = 0.78, SE = 0.08, *p* < .001, and baseline drug use, *b* = −0.58, SE = 0.29, *p* = .046, also remained significant predictors. Race was also significantly associated with alcohol use severity, *b* = −3.66, SE = 1.18, *p* = .003. The remaining psychopathy facets, age, sex, PTSD, and history of head injury were not significant predictors, *p*s > .05.

## 4. Discussion

The present study prospectively examined whether the four psychopathy facets differentially predicted drug and alcohol use severity over a six-month follow-up period among adults recovering from a violent injury. Consistent with the multidimensional conceptualization of psychopathy (Hare, 2003; Hare & Neumann, 2008; Neumann et al., 2007), the affective and antisocial facets demonstrated distinct prospective associations with substance-related outcomes despite substantial overlap among the four psychopathy dimensions. Specifically, affective psychopathic traits were uniquely associated with greater drug use severity at six-month follow-up, whereas antisocial psychopathic traits were uniquely associated with greater alcohol use severity after accounting for demographic characteristics, PTSD, history of head injury, baseline drug and alcohol use, and the remaining psychopathy facets. Although the association between psychopathy and substance misuse has traditionally been interpreted within a shared externalizing framework (Ellingson et al., 2018; Verona et al., 2018), the present findings extend this literature by demonstrating that distinct psychopathy facets may confer differential vulnerability to specific forms of substance misuse over time. By examining drug- and alcohol-related outcomes separately and simultaneously modeling the four psychopathy facets, the present findings provide evidence that prospective associations between psychopathy and substance misuse may differ across psychopathy dimensions and substance type.

Historically, the association between psychopathy and substance misuse has been conceptualized as reflecting a shared externalizing liability (Ellingson et al., 2018). From this perspective, alcohol and drug dependence, antisocial behavior, and impulsivity represent overlapping manifestations of a common externalizing spectrum, with the lifestyle and antisocial dimensions of psychopathy reflecting the psychopathic features most closely aligned with this liability (Ellingson et al., 2018; Rutherford et al., 2016; Verona et al., 2018). Consequently, the lifestyle and antisocial facets have generally been viewed as the primary drivers of the association between psychopathy and substance misuse, whereas interpersonal and affective traits have received comparatively less theoretical and empirical attention. This conceptualization is supported by evidence showing stronger associations between lifestyle and antisocial traits and substance misuse, as well as findings suggesting that the variance shared between psychopathy and externalizing psychopathology is largely attributable to the behavioral features of psychopathy rather than its interpersonal-affective characteristics (Brieman et al., 2024; Krueger et al., 2007; Patrick et al., 2005, 2013; Vincent et al., 2024). Against this backdrop, the present findings are noteworthy because affective psychopathic traits uniquely predicted greater drug use severity after accounting for baseline substance misuse and the substantial covariance among the psychopathy facets. These findings suggest that externalizing processes alone may not fully account for persistent drug-related problems among individuals with psychopathic traits and raise the possibility that affective psychopathic traits may confer risk for persistent drug use through processes that are at least partly distinct from the behavioral features of psychopathy.

The selective association between affective psychopathic traits and prospective drug use severity is theoretically informative because it implicates the dimension of psychopathy that contemporary neurobiological models conceptualize as the core impairment underlying the construct, providing a useful framework for understanding why affective psychopathic traits may uniquely confer vulnerability for persistent drug-related problems. Psychopathy has been conceptualized as arising, in part, from dysfunction within neural systems supporting the processing of affective information, particularly the amygdala and ventromedial prefrontal cortex, resulting in impaired stimulus-reinforcement learning and a diminished capacity for distress and punishment cues to guide subsequent behavior (Blair, 2006, 2007, 2008, 2013, 2019). Rather than reflecting a generalized impairment in emotional experience, this account emphasizes abnormalities in learning the affective value of actions and outcomes, limiting the extent to which adverse consequences acquire sufficient motivational significance to guide future decision-making. Consistent with this conceptualization, evidence from experimental studies and meta-analytic research indicates that individuals with psychopathic traits show impaired learning from painful aversive outcomes, alongside broader reductions in responsiveness to others’ distress and emotional cues (Atanassova et al., 2024; Burghart & Mier, 2022). Importantly, emerging facet-level psychophysiological evidence provides more specific support for altered aversive responding among individuals high in affective psychopathic traits. When the four psychopathy facets were examined simultaneously, higher affective traits were uniquely associated with dampened sympathetic nervous system reactivity during explicit fear induction, indicating reduced physiological responsivity to aversive emotional stimuli (Thomson, 2022). Collectively, these findings suggest that the affective facet may identify individuals for whom threatening, distressing, or otherwise aversive information carries diminished affective significance. In the context of drug use, diminished affective sensitivity to the physical, psychological, and interpersonal consequences of continued use may reduce the likelihood that these consequences elicit the emotional and motivational responses necessary to constrain subsequent behavior, providing one potential explanation for the prospective association observed in the present study. Individuals with elevated affective psychopathic traits may therefore be less likely to modify drug use in response to accumulating adverse consequences. However, reduced affective responsivity is not inherently substance-specific and, by itself, does not explain why affective psychopathic traits prospectively predicted drug-related, but not alcohol-related, severity.

The specificity of the association with drug use severity may reflect the distinctive environmental context in which persistent drug use occurs. Although both drug and alcohol misuse are associated with substantial health, interpersonal, and social consequences, illicit drug use may more frequently occur in contexts characterized by acute health risks, social marginalization, and criminal-legal involvement (Park et al., 2020; Scher et al., 2023; Strang et al., 2020; van Draanen et al., 2020; Volkow, 2021). These contexts may expose individuals to repeated and more immediate adverse consequences, including overdose, infectious disease, family disruption, financial instability, and legal consequences. In contrast, alcohol misuse is more often embedded within socially normative contexts, which may allow problematic patterns of use to develop or persist without the same degree of risk for immediate or externally imposed consequences, despite the substantial adverse consequences that can accompany severe alcohol-related problems. This distinction may be particularly relevant to the affective learning processes implicated in psychopathy (e.g., Blair, 2007, 2019). If adverse outcomes exert less affective and motivational influence among individuals with elevated affective psychopathic traits, repeated drug-related harms and sanctions may be less effective in modifying subsequent behavior, allowing use to persist despite accumulating adverse consequences. Thus, the differential association observed in the present study may reflect, in part, an interaction between affective psychopathic traits and differences in the contexts in which drug- and alcohol-related problems develop and persist, whereby deficits in punishment-related learning become particularly consequential when adaptive behavioral change depends on learning from repeated and immediate adverse outcomes (e.g., Blair, 2007, 2019). Although speculative, this interpretation provides one potential explanation for why affective psychopathic traits prospectively predicted drug use severity but not alcohol use severity.

It is also possible that the unique association between affective psychopathic traits and subsequent drug-related problems reflects differences in personality characteristics associated with problems involving different substances. Distinct personality profiles have been observed among individuals with drug- and alcohol-use disorders, and trait tendencies may interact with contextual factors to shape the type and course of substance-related problems (Zilberman et al., 2018). This possibility is particularly relevant to psychopathy, as interpersonal and affective traits may confer different vulnerabilities depending on the substance involved. Prior research has found that associations vary between psychopathy facets and substances. In a sample of incarcerated men, Walsh et al. (2007) found that the interpersonal facet was uniquely associated with cocaine dependence, whereas the Affective facet was inversely associated with cannabis dependence after accounting for the other psychopathy facets. Similarly, Brieman et al. (2024) identified substance-specific associations involving the interpersonal and affective facets across two independent correctional samples. Interpersonal traits were uniquely associated with cocaine-related outcomes, whereas Affective traits showed a negative association with substance-related outcomes that varied across alcohol and illicit substances (Brieman et al., 2024). Together, these findings suggest that associations between psychopathy facets and substance-related outcomes may vary as a function of individual differences and the substance involved, providing another possible explanation for why Affective psychopathic traits were associated with subsequent drug-related, but not alcohol-related, problems in the present study.

Differences in methodology between the present study and prior research may also help to account for variation in observed facet–substance associations. Prior studies have primarily examined incarcerated or correctional populations and assessed substance involvement or lifetime substance use disorder symptom counts using correlational designs. In contrast, the present study examined adults recovering from a violent injury and prospectively assessed six-month drug- and alcohol-related problem severity while controlling for baseline substance-related problems and shared variance among psychopathy facets. Differences in the populations studied, substance-related outcomes assessed, and study designs may therefore contribute to the divergent pattern of findings across studies. In particular, the present study’s prospective design and focus on subsequent substance-related severity may capture associations that are not evident when substance involvement or lifetime symptom counts are assessed cross-sectionally. These methodological and sample differences should therefore be considered when interpreting the unique association between affective psychopathic traits and subsequent drug-related severity observed in the present study. In sum, the association between affective psychopathic traits and subsequent drug-related problems may reflect contextual differences, variation in personality profiles, methodological differences, or an interplay among these factors.

The prospective association between antisocial psychopathic traits and alcohol misuse severity is broadly consistent with externalizing accounts of the psychopathy–substance misuse comorbidity. Antisocial psychopathic traits have been associated with alcohol misuse and a reduced likelihood of abstinence in prior cross-sectional research (Brieman et al., 2024; Walsh et al., 2007). Extending this literature, antisocial psychopathic traits uniquely predicted alcohol use severity after accounting for baseline alcohol involvement and shared variance among the four psychopathy facets in the present study. Thus, the relevance of antisocial psychopathic traits may extend beyond an increased propensity for alcohol involvement to the persistence of alcohol-related problems over time. This finding is consistent with the early-emerging and persistent externalizing pattern captured by the antisocial facet and with developmental evidence linking persistent externalizing psychopathology to subsequent alcohol misuse (Edwards et al., 2016; Lansford et al., 2023).

At the same time, the present findings diverge from previous cross-sectional research in which lifestyle psychopathic traits demonstrated associations with alcohol dependence (Brieman et al., 2024; Walsh et al., 2007). Although lifestyle traits were associated with six-month alcohol use severity at the bivariate level, this association was no longer significant after accounting for baseline substance misuse and covariation among the psychopathy facets, whereas antisocial traits emerged as the unique psychopathy predictor. This pattern may indicate that lifestyle-related features are associated with concurrent substance-related problems but do not uniquely account for subsequent alcohol-use severity beyond preexisting problems and shared variance with other psychopathy dimensions. Thus, the present findings provide partial support for the hypothesized role of the behavioral facets and suggest that the psychopathic traits associated with alcohol misuse cross-sectionally may not necessarily be those that uniquely contribute to the persistence of alcohol-related problems over time. Differences between the present study and prior research in sample characteristics, substance-related outcomes, and study design may also contribute to this divergent pattern. Future longitudinal research is needed to clarify whether the psychopathy facets differentially contribute to the initiation, persistence, and progression of alcohol-related problems.

### 4.1. Clinical Implications

The present findings may also have implications for the assessment and treatment of substance-related problems among individuals with elevated psychopathic traits. The association between affective psychopathic traits and subsequent drug-related problems suggests that affective characteristics may be relevant to understanding individual differences in the persistence of drug-related problems. Contemporary neurobiological accounts of psychopathy suggest that affective psychopathic traits are characterized by impairments in affective learning that reduce the extent to which adverse consequences modify subsequent behavior (e.g., Blair, 2008, 2019; Thomson, 2022). Taken together, this raises the hypothesis that the effectiveness of substance use interventions may vary as a function of affective psychopathic traits, particularly for approaches that depend on the motivational impact of naturally occurring consequences. This hypothesis could be tested by examining whether affective psychopathic traits moderate treatment responses across interventions that differ in their use of immediate versus delayed reinforcement. For example, contingency management providing explicit and immediate reinforcement for substance-related behavior change may represent one approach for testing whether reinforcement-based interventions are differentially effective across levels of affective psychopathic traits because it relies less on the affective and motivational significance of naturally occurring adverse consequences to shape behavior. Whether affective psychopathic traits moderate treatment response to different substance use interventions represents an important direction for future research.

### 4.2. Strengths and Limitations

Some limitations should be considered when interpreting the present findings. First, psychopathy was assessed using the SRP-SF, a self-report measure that may be subject to response biases, including impression management and limitations in self-awareness. Although the SRP-SF demonstrated good internal consistency in the current sample, self-report assessment may not fully capture the multidimensional nature of psychopathy. Future research should consider incorporating multimethod assessments of psychopathy to address potential limitations associated with self-report measurement. An additional consideration is the collinearity among the psychopathy facets (VIFs up to 4.32). Although these values were below commonly used thresholds for multicollinearity, the facet-level coefficients should be interpreted with caution regarding the partitioning of shared variance. Second, although the prospective design extends previous research based largely on cross-sectional and lifetime indices of substance misuse, the six-month follow-up period precludes conclusions regarding the longer-term course of drug- and alcohol-related problems. The six-month reference period used at follow-up also represents an adaptation of the standard administration of these measures, which typically assess substance-related problems over 12 months. The six-month timeframe was used to capture substance-related problems occurring during the prospective follow-up interval and reflects a different reference period than the 12-month baseline assessment. Studies incorporating repeated assessments over longer follow-up periods are needed to determine whether the observed facet-specific associations persist across time and whether psychopathy facets differentially predict the maintenance and progression of substance-related pathology. Third, participants were violently injured patients recruited following hospitalization for traumatic injury and therefore represent a high-risk population characterized by elevated exposure to violence and other forms of psychosocial adversity. Although this population is particularly relevant to the study of psychopathy and substance misuse, generalizability may be limited. Replication across clinical and community samples is needed to establish the generalizability of the present facet-specific findings. Finally, drug use severity was examined as a broad outcome, precluding examination of potentially meaningful differences across specific classes of drugs. Given the substantial heterogeneity in pharmacological effects, reinforcement processes, and adverse consequences associated with different substances, future research should investigate whether the prospective association between affective psychopathic traits and drug use severity generalizes across drug classes or is particularly pronounced for specific forms of drug-related pathology. Such work will be important for clarifying the conditions under which distinct psychopathic traits contribute to the course of substance-related problems.

## 5. Conclusions

The present study extends the psychopathy literature by demonstrating that distinct psychopathic traits differentially predict drug- and alcohol-related severity following violent injury. Whereas affective psychopathic traits were uniquely associated with greater drug use severity, antisocial psychopathic traits were uniquely associated with greater alcohol use severity, after accounting for demographic characteristics, psychiatric and injury-related covariates, baseline substance use, and shared variance among the remaining psychopathy facets. These findings suggest that multiple psychological pathways may contribute to persistence of substance-related pathology. More broadly, the findings underscore the value of the four-facet model for advancing understanding of heterogeneity within psychopathy and highlight the importance of examining specific psychopathic dimensions, rather than global psychopathy scores alone, when investigating clinically meaningful outcomes. Elucidating the distinct mechanisms linking individual psychopathy facets to substance misuse may ultimately inform more precise etiological models and facilitate the development of more targeted prevention and intervention strategies for individuals at the greatest risk of persistent substance-related harm.

## Figures and Tables

**Table 1:** Means, Standard Deviations, and Correlations Among Study Variables.

Variable	M	SD	1	2	3	4	5	6	7	8	9	10	11	12	13
1. Age	33.91	13.44	-												
2. Sex	0.36	0.48	−.15	-											
3. Race	0.79	0.41	−.04	−.18	-										
4. PTSD	0.40	0.49	−.05	.08	−.08	-									
5. Head Injury	0.40	0.49	.01	.19	−.20	.17	-								
6. Interpersonal	12.05	5.42	−.09	−.23 *	.00	.20	−.01	-							
7. Affective	14.74	5.27	−.23 *	−.13	.11	29 **	−.13	.69 ***	-						
8. Lifestyle	14.01	5.99	−.25 *	−.19	−.08	.39 ***	.03	.72 ***	.73 ***	-					
9. Antisocial	15.23	5.67	−.14	−.28 *	.10	.12	−.01	.76 ***	.65 ***	.68 ***	-				
10. Drug use (T1)	1.30	1.71	.02	−.27 *	.05	.02	−.14	.22	0.17	.27 *	.29 **	-			
11. Alcohol use (T1)	7.64	7.23	.00	−.01	−.11	.31 **	.21	.23 *	.21	.47 ***	.07	.01	-		
12. Drug use (T2)	1.31	1.88	−.01	−.23 *	−.11	.25 *	.06	.41 ***	.43 ***	.35 **	.41 ***	.31 **	.08	-	
13. Alcohol use (T2)	6.94	6.87	−.12	.15	−.27 *	.31 **	.17	.19	.19	.35 **	.14	−.14	.75 ***	.06	-

*Note. p* < .05 *, *p* < .01 **, *p* < .001 ***. PTSD = posttraumatic stress disorder.

**Table 2:** Multiple Linear Regression Predicting Six-Month Drug Use Severity.

Predictor	*b*	*SE*	*β*	*R* ^2^
Age	0.01	0.01	.07	.41
Sex	−0.58	0.36	−.14	
Race	−0.81	0.44	−.16	
PTSD	0.47	0.36	.12	
Head Injury	0.53	0.36	.13	
Baseline Drug use	0.39 ***	0.09	.39	
Baseline Alcohol Use	−0.03	0.03	−.11	
Interpersonal	0.03	0.05	.09	
Affective	0.12 *	0.05	.35	
Lifestyle	−0.01	0.06	−.04	
Antisocial	−0.01	0.05	−.02	

*Note. p* < .05 *, *p* < .001 ***. PTSD = posttraumatic stress disorder.

**Table 3:** Multiple Linear Regression Predicting Six-Month Alcohol Use Severity.

Predictor	*b*	*SE*	*β*	*R* ^2^
Age	−0.06	0.04	−.12	.69
Sex	1.54	1.04	.11	
Race	−3.66 **	1.18	-.22	
PTSD	1.45	1.04	.11	
Head Injury	−1.29	1.01	−.09	
Baseline Drug use	−0.58 *	0.29	−.15	
Baseline Alcohol Use	0.78 ***	0.08	.82	
Interpersonal	−0.07	0.15	−.06	
Affective	0.03	0.14	.02	
Lifestyle	0.32	0.16	.28	
Antisocial	0.43 **	0.14	.36	

*Note. p* < .05 *, *p* < .01 **, *p* < .001 ***. PTSD = posttraumatic stress disorder.

## Data Availability

The data presented in this study are available on request from the corresponding author.
